# Sleep in a warming world: why climate change demands a new sleep science agenda

**DOI:** 10.1093/sleep/zsag112

**Published:** 2026-05-29

**Authors:** Michael A Grandner

**Affiliations:** Sleep and Health Research Program, Department of Psychiatry, University of Arizona College of Medicine – Tucson, Tucson, AZ, United States

Sleep occurs in context. The physiology of sleep includes processes that involve nearly every system in the body, with effects on cardiovascular, metabolic, immune, cognitive, emotional, and behavioral health [1]. Yet, these physiologic processes do not occur in isolation; individuals sleep in a place, with a posture, after engaging in a set of behaviors that are at least partially environmentally determined [2]. The context of sleep has been explored as an important driver of sleep health—an upstream factor that may play a key role in our understanding of how sleep-related mechanisms impact health outcomes [3].

The role of climate as an environmental factor that contributes to the context of sleep has been discussed, and several prior studies have shown that sleep is at least somewhat related to weather patterns [4–6]. Further, experimental work shows that the thermoregulatory system plays key roles in sleep–wake regulation; thus, rising temperatures may exert an adverse impact on sleep [7–10].

In this issue of SLEEP, Minor *et al.* [11] suggest that a Global Climate and Sleep Task Force is needed. The paper argues that human sleep is influenced by ambient temperature and that it is possible that climate change is at least partially responsible for some of the societal-level loss in sleep that has been seen on a global scale. The paper points to a few key problems with the current literature and critical gaps that must be addressed in order to better understand these relationships. Of note, given the global impact of these problems and the need for global data that might be better able to model climate change (rather than more localized data), the paper stresses the importance of international collaboration in these efforts. Further, the paper suggests that climate data should be embedded more frequently in sleep research and sleep data should be added to global efforts to study climate change, both as an indicator of population health and economic potential, and also as a proximal health factor that is influenced by changing temperature.

**Figure 1:**
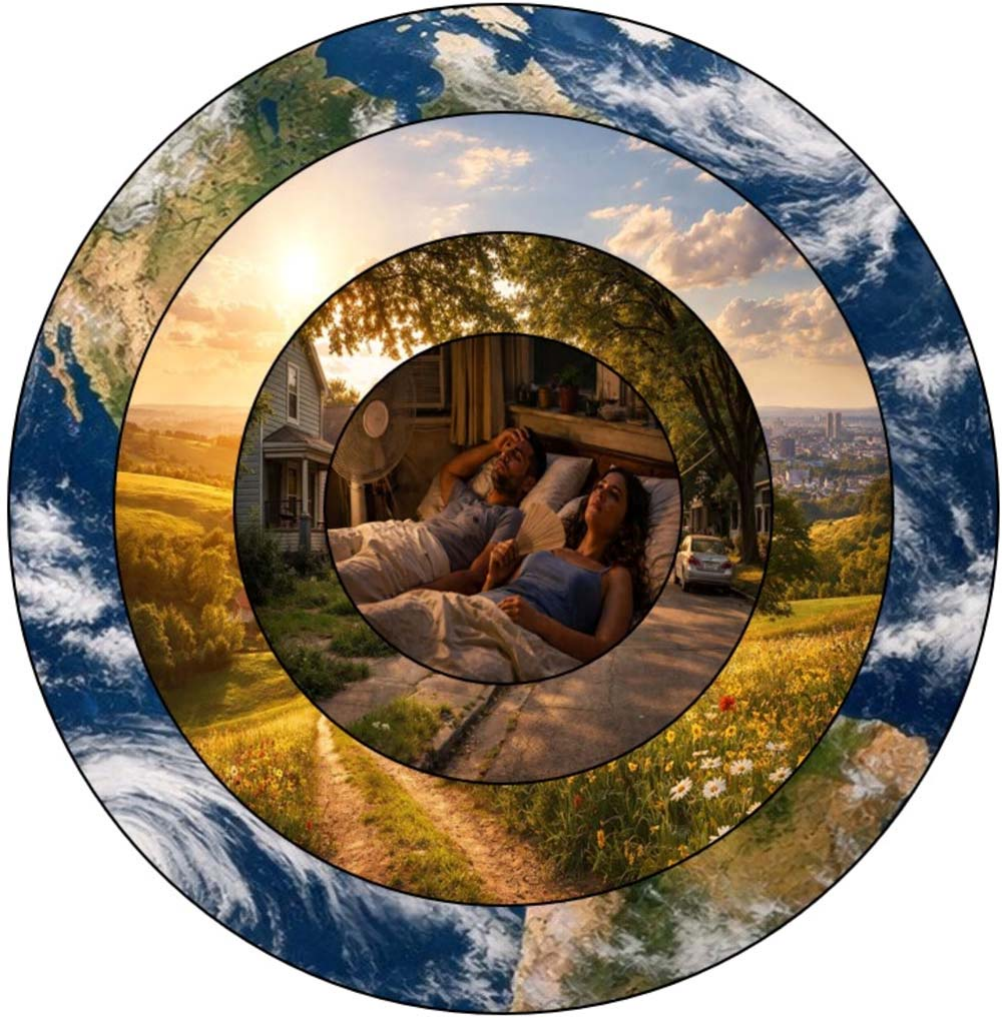
Graphical representation of how climate impacts sleep in a social-ecological framework. Global climate creates the context for regional climate, which itself drives the climate experience of specific neighborhoods, which represents the conditions that individuals need to manage regarding their own bedrooms. Difficult sleeping conditions in the bedroom impact the experience of sleep, which may have downstream impacts on health and well-being.

This conceptualization of sleep in relation to climate is described within the social-ecological model of sleep health [2, 3]. In this model, illustrated in Figure 1, climate change is represented as a societal-level factor that influences embedded social-level factors. For example, climate change may influence bedrooms, neighborhoods, home electricity use, and other factors, which themselves would influence individual-level factors such as decisions about when to go to bed and strategies to be able to sleep in a cool-enough environment. The model further describes how this layered influence links a global issue like climate change to individual health and mortality outcomes through changes that influence an individual’s sleep.

The model also suggests that interventions can occur at any level. At the societal level, sleep-friendly interventions can respond to climate change by enacting public policy and advocacy initiatives to not only combat rising temperatures but also incentivize energy policies, building practices, and commercial products that help protect the climate and/or help people keep cool during sleep. Social-level interventions could include industry-level policies, neighborhood-level home-building practices, and electricity usage practices that take positive steps to address climate change. For example, tax incentives for installing ceiling fans, double-paned windows, and solar panels may exert influence at the societal level. Interventions at the social level could include the development and dissemination of resources, products, and strategies to assist individuals as they try to keep their sleep environment cool in an environmentally safe way. For example, a neighborhood can install solar panels to improve energy utilization and promote building codes that prioritize energy-efficient windows. Some examples of individual-level interventions could include strategies to keep an individual’s bedroom cool. For example, an individual may choose to use a device that helps cool their sleeping surface rather than cooling all the air in a home with air conditioning.

Despite the proliferation of such products, there is a distinct lack of unbiased data published on the utility of energy-efficient strategies for controlling temperature. For example, there is a lack of studies that systematically evaluate the benefits of cooling mattress pads [12], cooling wearables [13], cooling bedding [14], and climate-friendly building cooling systems [15] on sleep in the context of energy utilization. This results in a lack of evidence upon which to base recommendations for keeping a bedroom cool, which can promote sleep health.

In addition to intervention studies, other research initiatives naturally arise from this wake-up call. As laboratory studies have extensively studied thermoregulatory control and sleep, less work has made sense of the emerging ubiquity of temperature sensors in many wearable devices [16]. Although skin temperature is generally unrelated to core body temperature, not only is there evidence that core body temperature might be unmasked from surface temperature [17], but there is emerging evidence that surface temperature itself may be a useful sleep metric [18, 19]. More work is needed to make optimal use of the technology that is already available at scale.
